# Knowledge Regarding Blood Pressure Measurement among First and Second Year Undergraduate Medical Students: A Descriptive Cross-sectional Study

**DOI:** 10.31729/jnma.7413

**Published:** 2022-03-31

**Authors:** Shavana Rajya Laxmi Rana, Bikalp Thapa, Lee Budhathoki, Yesha Shree Rajaure, Bipin Kumar Shrestha, Barun Mahat, Sunil Dhungel, Tara Man Amatya

**Affiliations:** 1 Department of Physiology, Nepalese Army Institute of Health Sciences, Kathmandu, Nepal; 2 Department of Community Medicine, Nepalese Army Institute of Health Sciences, Kathmandu, Nepal

**Keywords:** *blood pressure*, *knowledge*, *medical students*

## Abstract

**Introduction:**

Blood pressure measurement has a great implication in medicine. Every medical personnel should have a sound knowledge regarding blood pressure measurement. This study was conducted with the aim of determining the knowledge regarding blood pressure measurement among first and second year medical students of a medical college.

**Methods:**

A descriptive cross-sectional study was conducted by Department of Physiology of Nepalese Army Institute of Health Sciences from June to December 2021 after receiving the ethical approval from Institutional Review Committee with registration number 394. One hundred ninety-seven students from first and second year were included in the study using whole sampling technique. Data was entered and analysed using Microsoft Excel 2019 and Statistical Package for the Social sciences version 16.0. Descriptive statistics like frequency, percentage for binary data and mean, standard deviation for continuous data were calculated.

**Results:**

Among 197 students from first and second year, 175 (88.83%) had satisfactory knowledge regarding blood pressure measurement with score ≥8 in Objective Structured Practical Examination. Eighty five (85.86%) students from first year and 90 (91.84%) students from second year had scores ≥8, hence had satisfactory knowledge on blood pressure measurement. A total of 18 (18.18%) first year students achieved a score of 10, while 24 (24.49%) students of the second year scored 12, which were the modal score.

**Conclusions:**

The proportion of first and second year students having satisfactory knowledge regarding blood pressure measurement is higher in our study.

## INTRODUCTION

Blood pressure (BP) measurement is an important medical skill. Hypertensive heart diseases being major contributor towards global morbidity and mortality, it plays an important role in identifying individuals as having BP related risk and in management.^1,2^ Manual measurement of BP requires psychomotor skill, attention in visual and auditory aspects and adherence to the recommended technique.^1,3–5^

The sole act of BP measurement is not the only thing that reflects accurate BP of the subject. Position of the subject, recent exertion, consumption of caffeine, smoking, full bladder etc. also have influence over BP.^1^ An accurate measurement of BP not only requires practical skill, but also the knowledge about factors that have influence over BP. There are few literatures available on the knowledge regarding theory and skill based activity of BP measurement among medical students.

This study aims to determine the knowledge regarding BP measurement among first and second year undergraduate medical students of a medical college.

## METHODS

This is a descriptive cross-sectional study conducted in first and second-year medical students of Nepalese Army Institute of Health Sciences (NAIHS) in the Department of Physiology between June to December 2021. Ethical approval for the study was given by the Institutional Review Committee (IRC) of NAIHS dated June 2021 with registration number 394. Whole sampling technique was used.

All first and second year MBBS students of NAIHS who agreed to take part in the study were included in the research. Absentees during the tutorial session of practical class regarding the measurement of blood pressure and second year students with less than 50% attendance in theory classes of cardiovascular physiology or those who were absent specifically in theory classes related to blood pressure were excluded. A total of 197 students (99 students from first year MBBS and 98 students from second year) MBBS qualified for this study. One student from first year, and two students from second year were absent during the practical tutorial and demonstration session and were excluded from the study.

The first year MBBS students were tutored and demonstrated the method of measurement of blood pressure in humans in accordance with the AHA scientific statement.^1^ They had not studied the theory portion of cardiovascular physiology as per MBBS TU curriculum. The second year MBBS students studied the theoretical portions of cardiovascular physiology, followed by a practical tutorial and demonstration on the method of measurement of blood pressure in humans. Following the practical tutorial and demonstration to the two groups, considering the inclusion and exclusion criteria, and after a week an OSPE (Objective Structured Practical Examination) was conducted. Healthy middle aged male with no known comorbidities were taken as subjects for the OSPE. The students were given a scenario mentioning the subject's condition and background, and were assessed for history taking, rest before measurement, proper position of the subject, cuff positioning and tying, palpatory method performance and timing, auscultatory method performance and reporting of the result. The OSPE consisted of all the necessary steps required for accurate measurement of BP, which could broadly be segregated into knowledge based activity, and skill based activity. The knowledge based activity included relevant history taking, proper patient positioning, selection of proper size BP cuff etc, while the skill based activity included proper tying of the cuff, slow releasing of cuff pressure etc. The students were evaluated based on their performance on OSPE by an evaluator who had conducted the practical tutorials and demonstrations. The knowledge regarding BP measurement among the study participants was considered to be satisfactory if the score obtained on the OSPE was more than or equals to 8.

Data was entered and analysed using Microsoft Excel 2019 and Statistical Package for the Social sciences (SPSS) version 16.0. Descriptive statistics like frequency, percentage for binary data and mean, standard deviation for continuous data were calculated.

## RESULTS

Out of 197 study participants, 175 (88.83%) students had satisfactory knowledge regarding BP measurement (i.e. score obtained during OSPE ≥8). Eighty-five (85.86%) students from first year and 90 (91.84%) students from second year had scores ≥8 in OSPE, hence had satisfactory knowledge on BP measurement.

A total of 18 (18.18%) first year students achieved a score of 10, while 24 (24.49%) students of the second year scored 12, which were the modal score (Table 1).

**Table 1:** Scores obtained by the study participants during OSPE.

Scores obtained	First Year n (%)	Second Year n (%)
0	-	-
1	-	-
2	-	-
3	-	-
4	1 (1.0)	-
5	-	1 (1.0)
6	6 (6.1)	4 (4.1)
7	7 (7.1)	3 (3.1)
8	17 (17.2)	3 (3.1)
9	17 (17.2)	5 (5.1)
10	18 (18.2)	15 (15.3)
11	13 (13.1)	9 (9.2)
12	15 (15.2)	24 (24.5)
13	4 (4.0)	20 (20.4)
14	1 (1.0)	14 (14.3)
15	-	-

While answering the knowledge based activities during OSPE, all the students from first year 99 (100%) and second year 98 (100%) believed that selecting proper cuff size is essential for BP measurement (Table 2).

**Table 2:** Knowledge based activities during OSPE on BP measurement.

Knowledge based activities	First year n (%)	Second year n (%)
Takes relevant history	4 (4.0)	19 (19.4)
Ensures rest	9 (9.1)	61 (62.2)
Proper body position	48 (48.5)	60 (61.2)
Proper arm position	77 (77.8)	71 (72.4)
Selects proper cuff size	99 (100.0)	98 (100.0)
Checks zero error in instrument	74 (74.7)	93 (94.9)
Cuff inflation to proper level	44 (44.4)	66 (67.3)

Similarly, for the skill based activities during OSPE, majority of the students from first year 91 (91.9%) believed that tying cuff securely is important and majority of the students from second year 96 (98%) reported BP properly (Table 3).

**Table 3:** Skill based activities during OSPE on BP measurement.

Skill based activities	First year n (%)	Second year n (%)
Ties cuff securely	91 (91.9)	86 (87.8)
Ensures proper cuff position	74 (74.7)	84 (85.7)
Ties exposing cubital fossa	45 (45.5)	59 (60.2)
Palpates radial artery	65 (65.7)	85 (86.7)
Does palpatory method	59 (59.6)	66 (67.3)
Auscultate over brachial artery	87 (87.9)	85 (86.7)
Slow cuff deflation	90 (90.9)	83 (84.7)
Reports BP properly	81 (81.8)	96 (98.0)

An increase in the mean scores can be seen in second year students in all the components of the OSPE-overall score, knowledge and skill components (Table 4).

**Table 4:** Mean OSPE score obtained by the first and the second year medical students.

	Maximum possible score	First year score Mean±SD (n= 99)	Second year score Mean±SD (n= 99)	Percentage increase of score in second year students (%)
Overall Score	15	9±1.98	9.8±2.19	5.33
Knowledge Component Score	7	3.58±1.12	4.77±1.38	17.00
Skill Component Score	8	5.98±1.41	6.57±1.25	7.37

## DISCUSSION

Burgess SE, et al. has shown how proper blood pressure measurement has major implications in treatment decisions and how failure to follow precautions during blood pressure measurements can give wrong blood pressure readings during usual care with BP readings being lower when American Heart Association recommendations were followed while measuring the BP.^6^ Our study showed a satisfactory result regarding the knowledge on BP measurement among first and second year medical students.

Our results indicate that second year MBBS students with a background theoretical knowledge of cardiovascular system adhered to the most steps of manual blood pressure measurement and took necessary precautions. The higher overall, as well as the knowledge and skill scores obtained by second year students could be attributed to the fact that the background knowledge helped them understand the importance of adhering to the guidelines and precautions during blood measurement as advised by the American Heart association.^1^ The presence of knowledge and demonstration of skill are complementary to each other. The students with theoretical knowledge probably understood the consequences of incorrect positioning of arms and body can have on the blood pressure as seen in studies by Adiyaman A, et al, Eser I, et al. and Mourad A, et al.^7–9^

A study by Brokalaki, et al. have shown that supplementary education in clinical tutoring influences the accuracy in clinical skill in nursing students of year one.^10^ Ballard, et al. showed that an extra hour of teaching significantly improved nurse's accuracy during BP measurement.^11^ This points towards the option that supplementary classes in BP measurement might benefit the first year MBBS students and these findings are in line with our study which shows better mean scores in second year students who have taken theory classes of cardiovascular physiology.

The strength of this study is that it was conducted as a part of a scheduled training program in a medical college of MBBS TU curriculum, evaluated by certified physiologists. Knowledge, skill and attitude are the three pillars of clinical competency. The OSPE designed in the study included the assessment of knowledge and the skill attributes required for the measurement of blood pressure. However, assessment of attitude viz. proper handling of instruments, demonstrating proper communication skills etc were not assessed as although they are necessary for medical practitioners, they directly do not affect the BP, nevertheless in a real scenario all of the three components determine the grade of doctor'patient interaction.

Since this study was conducted in single medical institution, the results of this study cannot be generalized to the population of total medical students in the country.

## CONCLUSIONS

The proportion of first and second year students having satisfactory knowledge regarding blood pressure measurement is higher in our study. It seems logical to teach blood pressure measurement skill in second MBBS students after a background knowledge of cardiovascular system for better understanding and adherence to the guidelines and this skill to be included in second year MBBS medical curricula. Alternatively, including BP measurement in both years of MBBS medical curriculum could also be beneficial as it would reinforce the learning, however it would increase the number of teaching hours, which could be considered as a necessary investment.
